# Efficacy and safety of everolimus in Chinese metastatic HR positive, HER2 negative breast cancer patients: a real-world retrospective study

**DOI:** 10.18632/oncotarget.16336

**Published:** 2017-03-17

**Authors:** Chengcheng Gong, Yannan Zhao, Biyun Wang, Xichun Hu, Zhonghua Wang, Jian Zhang, Sheng Zhang

**Affiliations:** ^1^ Department of Medical Oncology, Fudan University Shanghai Cancer Center, Department of Oncology, Shanghai Medical College, Fudan University, Shanghai, 200032, China

**Keywords:** everolimus, endocrine therapy, metastatic breast cancer, real-world study

## Abstract

**Background:**

Everolimus combined with endocrine therapy has been proved to be effective among postmenopausal women with hormone receptor-positive human epidermal growth factor receptor-2 negative (HR+/HER2-) metastatic breast cancer (MBC). We aimed to evaluate the efficacy and safety of everolimus plus endocrine therapy in Chinese real-world practice for the first time, and investigate factors associated with efficacy.

**Methods:**

Seventy-five HR+/HER2- MBC patients were included in this retrospective study who received everolimus plus endocrine therapy after progression on prior endocrine therapy in Fudan University Shanghai Cancer Center (FUSCC) between June 2013 and February 2016. Main outcome measures are progression free survival (PFS), overall survival (OS), objective response rate (ORR), clinical benefit rate (CBR) and safety profile.

**Results:**

After a median follow up of 10.3 (range: 2.1-32.2) months, median PFS was 5.9 months (95%CI 4.6-7.2), and median OS was not reached. The CBR was 38.8% (95%CI, 26.8-50.8) and ORR was 9.0% (95%CI, 2.0-16.0). Most common all-grade adverse events were stomatitis (57.1%), fatigue (25.7%), infection (24.3%) and hyperglycemia (21.4%). The most common ≥3 grade adverse events were stomatitis (9.3 %) and thrombocytopenia (5.7%). No treatment-related death was documented during and one month after the drug administration.

**Conclusions:**

The combination of everolimus and endocrine therapy proved to be effective in Chinese population. The safety profiles were similar to previous studies but incidences were lower. In conclusion, everolimus combined with endocrine therapy provides a reasonable option for Chinese HR+/HER2- metastatic breast cancer patients.

## INTRODUCTION

Breast cancer is one of the most prevalent malignancy and the leading causes of cancer morality in women worldwide and in China [1, 2]. Endocrine therapy (ET) is the cornerstone of the treatment for hormone-receptor positive (HR+) breast cancer (BC), accounting for approximately 70% of breast cancer [3–5]. However, patients receiving endocrine therapy eventually experience disease progression, either fail to respond in the first evaluation or progress after initial response [6]. Once the patients progress on initial endocrine therapy, sequential lines of ET offer limited clinical benefit. Emerging studies have been done to explain the mechanism behind endocrine resistance in order to identify new therapeutic strategies. The activation of phosphatidylinositol 3-kinase (PI3K)/Akt/ mammalian target of rapamycin (mTOR) pathway proved to be one of the key factors of endocrine resistance [7, 8]. Preclinical studies indicate that the inhibition of mTOR signaling has antitumor effect and can restore sensitivity to fulvestrant, letrozole, and tamoxifen in breast cancer cells [9–11].

Everolimus (Afinitor, Novartis), an orally selective inhibitor of mTORC1, has been shown to enhance the antitumor effect of endocrine therapy in breast cancers [12–15]. BOLERO-2 (Breast cancer trial of OraL EveROlimus- 2), a phase III study, demonstrated that the addition of everolimus to exemestane significantly prolonged the PFS from 3.2 months to 7.8 months (from 4.1 to 11.0 months by central review) in postmenopausal HR+/HER2- advanced breast cancer patients refractory to nonsteroidal aromatase inhibitor (NSAI) [16, 17]. Based on the impressive improvement on PFS, everolimus in combination with exemestane was approved for the treatment of postmenopausal HR+/HER2- advanced breast cancer patients recurring or progressing on prior NSAIs in the USA and European Union.

In China, however, the indication of everolimus in advanced breast cancer hasn't been approved, thus there is limited data about efficacy and safety of everolimus in Chinese breast cancer patients. Our study aimed to address this knowledge gap by providing first-hand evidence of the real-world efficacy and safety profile of everolimus in Chinese metastatic HR+/HER2- breast cancer patients.

## MATERIALS AND METHODS

### Patients

Patients with HR+/HER2- advanced breast cancer who failed on previous endocrine therapy and were treated with everolimus plus endocrine therapy in any line in metastatic setting from June 2013 to February 2016 in Fudan University Shanghai Cancer Center (FUSCC) were included. Data were retrospectively obtained from patients’ medical history. Everolimus combined with chemotherapy or target therapy were not discussed in this study. Demographic and clinical characteristics were evaluated in all patients. This study was approved by Fudan University Shanghai Cancer Center.

### Treatment

Everolimus was usually initiated at the dose of 10 mg or in some instances at 5 mg daily, according to patients’ tolerance and request. The treatment could be interrupted, reduced to 5 mg once daily, or permanently discontinued due to its adverse events. The endocrine therapy combined with everolimus was up to doctors’ choice based on prior endocrine treatments.

### Efficacy and safety

Efficacy was assessed by progression free survival (PFS), overall survival (OS), objective response rate (ORR), and clinical benefit rate (CBR). PFS was defined as time from initiation of everolimus to disease progression or death. OS was estimated from the date of treatment initiation to death of any cause or last follow-up. CBR was defined as the percentage of complete response (CR), partial response (PR) or stable disease (SD) for ≥24 weeks and ORR was considered as the percentage of CRs and PRs. Efficacy was evaluated by CT, MRI, bone scan and physical examination every 2-3 months until disease progression. Tumor responses were confirmed by researcher according to Response Evaluation Criteria in Solid Tumors (RECIST) 1.1 criteria.

Adverse events (AEs) were determined retrospectively based on patients’ medical records and laboratory tests results. Nonhematologic AEs were also evaluated by telephone follow-up. Patients without post-treatment safety evaluation were excluded from safety population. AEs were assessed according to the National Cancer Institute Common Terminology Criteria for Adverse Events version 4.0.

### Statistical analysis

Quantitative data are presented as median (range) or number of patients (percentage).

PFS and OS were estimated by the Kaplan- Meier method and the hazard ratios (HRs) and corresponding 95% confidence intervals (CIs) were estimated using the Cox proportional harzard model.

Exploratory univariate analyses were performed with the log- rank test using the following variables: age, disease-free interval (DFI), number of metastatic sites (1-2 vs ≥3), visceral metastases, liver/lung/bone metastases, lines of previous chemotherapy and endocrine treatment (0-1 vs ≥2), hormone resistance (primary vs secondary), the occurrence of symptomatic stomatitis, and combinational endocrine treatment. Primary endocrine resistance was defined as: a relapse during the first 2 years of adjuvant ET, or progress within first 6 months of first-line ET for MBC. Secondary endocrine resistance was defined as: a relapse during adjuvant ET but after the first 2 years, or a relapse within 12 months of completing adjuvant ET, or progress ≥6 months after initiating ET for MBC [18]. Symptomatic stomatitis was defined as showing symptoms of stomatitis, such as pain and functional impact.

Cox multivariate models were performed based on the univariate analyses results. All expressed P values and CIs were two tailed. The significance level of statistical tests was set at p < 0.05. CBR and ORR were calculated with their 95% CI. The safety population included patients who had post-treatment safety evaluation. AEs were summarized using percentages and frequency counts. The Kaplan–Meier method was used to evaluate time-to-onset of AEs of clinical interest. All statistical analyses were conducted using SPSS IBM® version 22.

## RESULTS

### Patients and treatment

A total of 75 HR+/HER2- metastatic breast cancer patients treated with everolimus plus endocrine therapy between June 2013 and February 2016 in FUSCC were included. Clinicopathological characteristics at the initiation of everolimus are shown in Table 1. The median age of the patients was 53 (range 24-73) years. Most patients were in postmenopausal status (80.0%). 13 (17.3%) premenopausal women were required to receive medical ovarian suppression during everolimus treatment. 5 patients were de novo stage IV breast cancer (6.7%). All patients were histologically confirmed HR+/HER2- advanced breast cancer patients, except that 5 patients’ HER-2 data were not available. More than half of the patients had ≥3 metastatic sites. The most common sites of metastases were bone (69.3%), lung (57.3%) and liver (46.7%). Most patients had visceral involvement (82.7%).

**Table 1:** Patients and tumor characteristics (*N* = 75)

Characteristics	Everolimus plus endocrine therapy	
	No.	%
**Age (years)**		
Median	53	
Range	24-73	
**Menopausal status**		
Postmenopausal	60	80.0
Premenopausal	13	17.3
Unknown	2	2.7
**Hormone receptor and HER-2 status**		
HR positive	75	100
HER-2 negative*	70	93.3
**de novo stage IV breast cancer** 5 6.7		
**Disease-free interval (months)**		
<12	9	12
12-24	14	18.7
>24	47	62.7
**No. of metastatic sites**		
1	9	12
2	21	28
≥3	45	60
**Metastatic sites**		
Lung	43	57.3
Liver	35	46.7
Bone	52	69.3
**Visceral disease**	62	82.7
**Lines of endocrine therapy**		
First line	8	10.7
Second line	31	41.3
Third or more line	36	48
**Previous endocrine therapy**		
Letrozole or anastrozole	68	90.7
Tamoxifen	50	66.7
Exemestane	31	41.3
Fulvestrant	29	38.7
**The most recent endocrine therapy**		
Letrozole or anastrozole	38	50.6
Fluvestrant	19	25.3
Exemestane	13	17.3
Tamoxifen	5	6.7
**Hormone resistance**		
Primary endocrine resistance	18	24
Secondary endocrine resistance	55	73.3
Unable to determine	2	2.7
**Previous chemotherapy**		
(Neo)adjuvant therapy only	17	22.7
Metastatic disease	58	77.3
**Lines of metastatic chemotherapy**		
1	16	21.3
2	17	22.7
≥3	25	33.3

* 5 patients’ HER-2 data were not available.

All patients had received previous endocrine therapy, including NSAIs (90.7%), tamoxifen (66.7%), exemestane (41.3%), and fulvestrant (38.7%). The most recent therapy before everolimus was NSAIs (50.6%). By the definitions previously described, primary resistance occurred in 24% of the patients while 73.3% had secondary resistance. 77.3% of the patients had received chemotherapy in metastatic settings.

At the cutoff time (May 25th, 2016), the combination was ongoing for six patients. Reasons for treatment discontinuation were disease progression (62.3%), intolerable toxicity (20.3%), patients’ choice (14.4%) and loss of follow up (2.9%). Median duration of everolimus treatment was 15.1 (range 1-131.6) weeks. Oncologists’ choices of concomitant endocrine therapy were as follow: exemestane (72%), NSAIs (14.7%), tamoxifen (8.0%), and fulvestrant (5.3%).

### Efficacy

**Figure 1:**
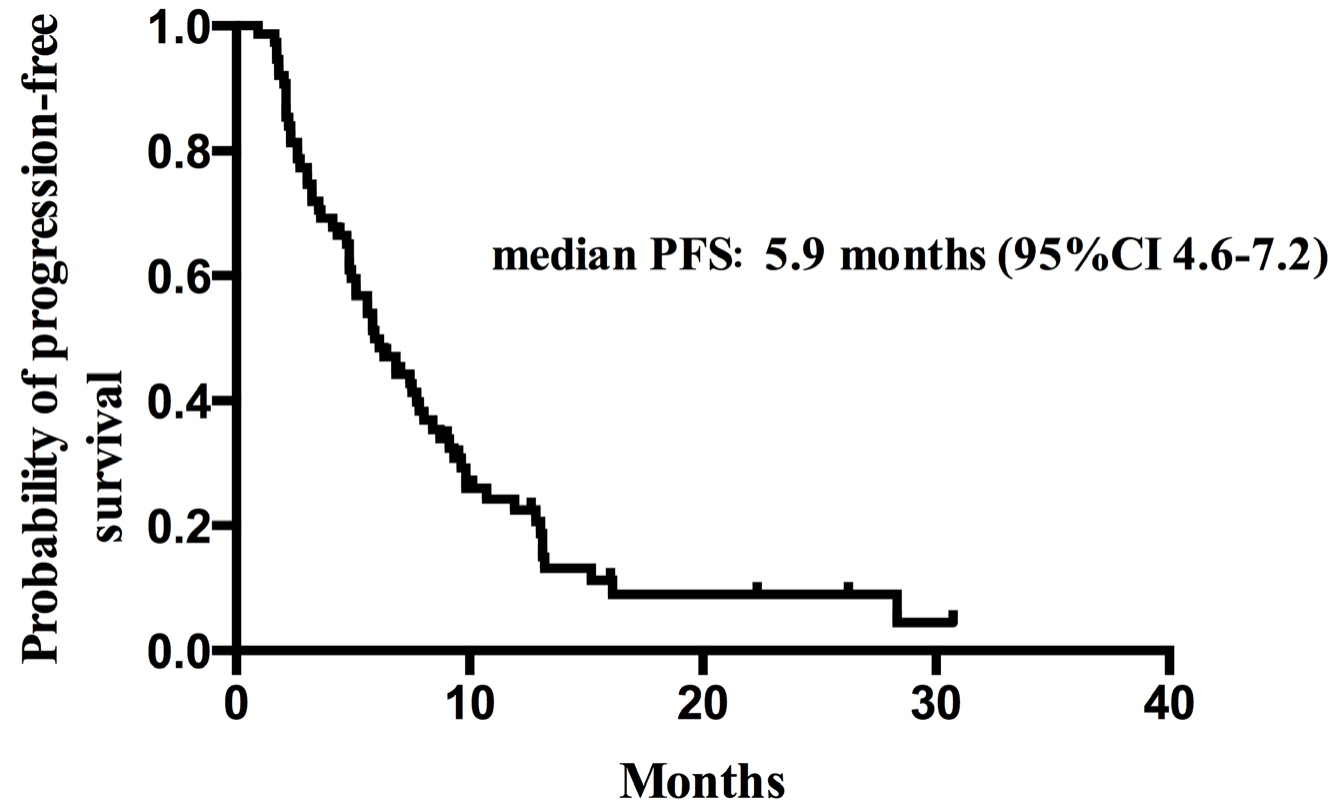
Kaplan–Meier estimates of progression-free survival of patients treated with everolimus and endocrine therapy

At a median follow up of 10.3 (range: 2.1-32.2) months, median PFS was 5.9 months (95%CI 4.6-7.2). With 22 (29.3%) patients died, overall survival data were immature at the time of the analysis. The Kaplan–Meier PFS is shown in Figure 1.

**Figure 2:**
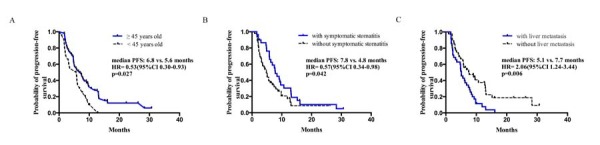
Kaplan–Meier curves for progression-free survival For patients stratified by potential factors related with PFS. **A**. Age, **B**. Symptomatic stomatitis. **C**. Liver metastasis. Abbreviations: CI, confidence interval; PFS, progression-free survival.

Tumor responses were shown in Table 2. 8 patients’ responses were unable to evaluate. Among 67 patients with evaluable disease, 6 patients (9.0%) achieved PR, 44 patients (65.7%) had SD, and 17 patients (25.4%) reported PD after the combinational treatment of everolimus and endocrine therapy. This resulted in a CBR of 38.8% (95% CI, 26.8-50.8) and an ORR of 9.0% (95% CI, 2.0-16.0). Univariate analysis indicated that PFS was significantly longer in patients ≥45 years old than in those <45 years old (HR, 0.53, 95%CI 0.30-0.93, *p* = 0.027), in patients with symptomatic stomatitis during treatment than in those without (HR,0.57, 95%CI 0.34-0.98, *p* = 0.042) and in patients without liver metastasis than those with liver metastasis (HR, 2.06, 95%CI 1.24-3.44, *p* = 0.006) (Table 3& Figure 2). Cox multivariate models were derived from the univariate analysis results. A significant association was observed by Cox multivariate analysis between PFS and the presence of liver metastasis (HR, 1.93; 95%CI 1.13-3.28, *p* = 0.016).

**Table 2:** Tumor responses (*N* = 67)

Responses	No. of patients	%
CR	0	0
PR	6	9.0
SD	44	65.7
PD	17	25.4
ORR(%)		9.0 (95%CI, 2.0-16.0)
CBR(%)		38.8 (95%CI 26.8-50.8)

Abbreviations: CR, complete response; PR, partial response; SD, stable disease; PD, progression disease; ORR, objective response rate; CBR, clinical benefit rate

**Table 3:** Exploratory analysis of factors to predict PFS of everolimus

Factors	PFS(months)	HRs	95%CI	*P* value
**Age (years)**^#^				
≥45 *N* = 55	6.83	0.53	0.30-0.93	0.027*
<45 *N* = 20	5.57			
**Menstruation status**^+^				
Postmenopausal *N* = 60	6.30	0.64	0.34-1.21	0.171
Premenopausal + OFS *N* = 13	5.83			
**DFI (months)**				
>24 *N* = 47	5.90	0.87	0.50-1.51	0.872
<24 *N* = 23	5.77			
**No. of metastatic sites**				
1-2 *N* = 30	6.30	0.94	0.57-1.57	0.820
≥3 *N* = 45	5.83			
**Visceral disease**				
Yes *N* = 62	6.10	1.27	0.64-2.51	0.495
No *N* = 13	5.90			
**Liver metastasis**^#^				
Yes *N* = 35	5.10	2.06	1.24-3.44	0.006*
No *N* = 40	7.70			
**Lung metastasis**				
Yes *N* = 43	6.83	0.82	0.50-1.35	0.430
No *N* = 32	5.63			
**Bone metastasis**				
Yes *N* = 52	5.57	1.65	0.94-2.89	0.082
No *N* = 23	9.07			
**No. of previous metastatic chemotherapy**^#^				
≥2 *N* = 42	5.57	1.55	0.92-2.62	0.098
0-1 *N* = 33	7.70			
**No. of previous metastatic endocrine therapy**				
≥2 *N* = 36	5.90	1.03	0.62-1.69	0.921
0-1 *N* = 39	5.77			
**Hormone resistance**^#+^				
Primary Resistant *N* = 18	5.13	1.21	0.66-2.23	0.532
Secondary Resistant *N* = 55	6.10			
**Symptomatic stomatitis**^#^				
Yes *N* = 30	7.80	0.57	0.34-0.98	0.042*
No *N* = 40	4.77			
**Combined endocrine therapy**				
Exemestane *N* = 54	6.77	NA	NA	NA
Tamoxifen *N* = 6	5.77			
Anatozole *N* = 6	4.77			
Letrozole *N* = 5	3.23			
Fulvestrant *N* = 4	3.0			

Abbreviations: HR, hazard ratio, CI, confidence interval, OFS, ovarian function suppression, DFI, disease-free interval.

*p < 0.05 is considered significant.

# factors included in Cox multivariate models.

+ Missing data were not included in the analysis.

### Safety

Safety analysis included 70 patients who had post-treatment safety evaluation after receiving the study treatment. Most frequent all-grade non-hematological side effects included stomatitis [Preferred terms of adverse events are used in this paper. The corresponding terminology used in CTCAE 4.0 are listed below. Stomatitis: mucositis oral (CTCAE 4.0). Noninfectious pneumonitis: pneumonitis. Thrombocytopenia: platelet count decreased. Leukopenia: white blood cell counts decreased. Hyperlipidemia includes cholesterol high and hypertriglyceridemia. Edema includes edema face, edema limbs and edema trunk. Rash includes rash pustular, rash acneiform, papulopustular rash and rash maculo-papular](57.1%), fatigue (25.7%), infection (24.3%), rash (18.6%), edema (14.3%), cough (12.9%), diarrhea (12.9%) and noninfectious pneumonitis (NIP) (10.0%). Most common laboratory abnormities were hyperglycemia (21.4%), alanine aminotransferase increased (18.6%), aspartate aminotransferase increased (18.6%), anemia (14.3%), hyperlipidemia (10.0%) and thrombocytopenia (10.0%). Most events were grade 1 or 2. The most common grade 3 events were stomatitis (9.3%) and thrombocytopenia (5.7%). No grade 4 events were observed. These findings are consistent with those from previous studies. No treatment-related death was documented during and one month after the drug administration. All adverse events are listed in Table 4.

**Table 4:** Adverse events (*N* = 70)

	Adverse Events	All grades (%)	≥ Grade 3 (%)
Non hematological events	Stomatitis	57.1	9.3
	Fatigue	25.7	1.4
	Infection	24.3	2.9
	Rash	18.6	0
	Edema	14.3	0
	Cough	12.9	0
	Diarrhea	12.9	1.4
	Noninfectious pneumonitis	10.0	0
	Pyrexia	10.0	0
	Anorexia	7.1	0
	Hypertension	7.1	2.9
	Weight loss	5.7	0
Biological events	Hyperglycemia	21.4	1.4
	ALT increased	18.6	1.4
	AST increased	18.6	1.4
	Hyperlipidemia*	10.0	0
Hematological events	Anemia	14.3	4.3
	Thrombocytopenia	10.0	5.7
	Leukopenia	5.7	0

Abbreviations: ALT, alanine aminotransferase, AST, aspartate aminotransferase. *Includes hypercholesterolemia (5.7%) and hypertriglyceridemia (4.3%).

**Figure 3:**
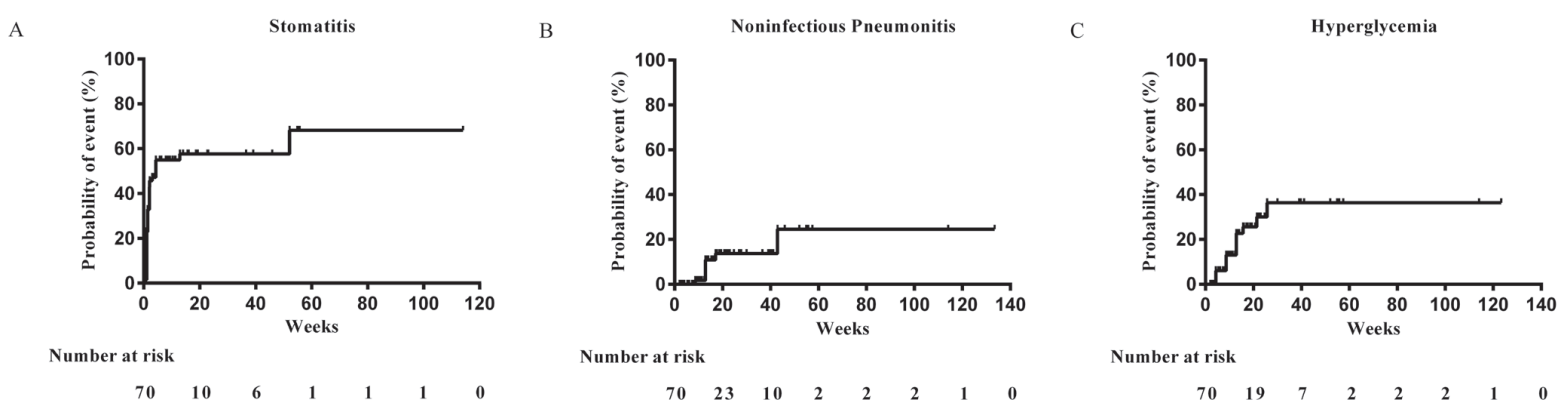
Cumulative risk estimates for initial onset of adverse events of clinical interest A. Stomatitis. B. Noninfectious pneumonitis. C. Hyperglycemia.

Further analysis revealed that more than 40% of all stomatitis events were reported in the first 2 weeks (cumulative risk, 42.9%). The median time of noninfectious pneumonitis onset was 13 weeks (cumulative risk, 10.9%). The most common laboratory abnormity was hyperglycemia. 21.4% patients had hyperglycemia during study. The median time of onset was 8.6 weeks (cumulative risk 13.0%). In most cases, the hyperglycemia was mild and controllable by oral hypoglycemic agent. One patient had grade 3 hyperglycemia and the dose of everolimus was reduced to 5 mg. Cumulative risk estimates for initial onset of stomatitis, noninfectious pneumonitis, and hyperglycemia were shown in Figure 3.

**Figure 4:**
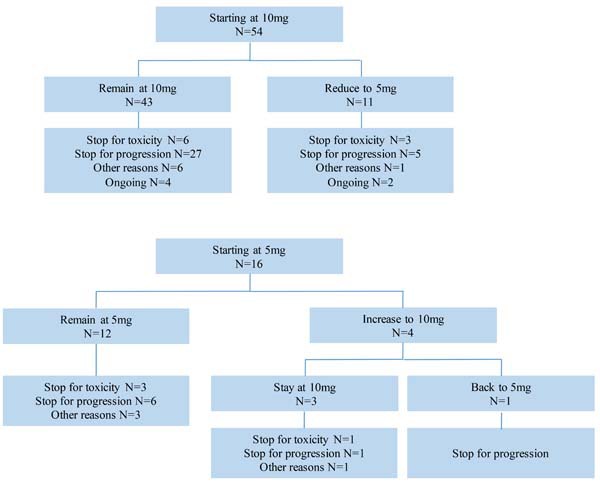
Dose pattern of everolimus

Everolimus was initially prescribed at the standard dose of 10 mg daily in 54 patients (77.1%) and at 5 mg daily in 16 patients (22.9%). In patients who initialed the treatment at 10mg, 11 patients were reduced to 5 mg during the treatment. Among patients who began at 5 mg daily, 4 patients increased to 10 mg after the first few weeks while 12 patients remained at 5 mg along the treatment (see Figure 4).

The most common AEs leading to dose interruptions/reductions in our study is stomatitis (N = 7), infection (*N* = 6), thrombocytopenia (*N* = 4), alanine/aspartate aminotransferase increased (*N* = 3), rash (*N* = 2), edema (*N* = 2) and NIP (*N* = 2). Treatment discontinuations because of AEs were 18.6% patients.

## DISCUSSION

The combination of everolimus and endocrine therapy has been recommended for postmenopausal HR+/HER2- breast cancer patients in NCCN guideline. However, the registering clinical trials of everolimus in breast cancer hasn't started yet in China. Little evidence exist regarding the effectiveness and safety of this combination in Chinese patients. This study reported the efficacy of everolimus based endocrine therapy in Chinese population for the first time. In this study of HR+/HER2- metastatic breast cancer, everolimus combined with endocrine therapy led to a PFS of 5.9 months (95%CI 4.6-7.2). And the median OS was still not reached with a median follow up of 10.3 months. The combination of everolimus and endocrine therapy in the present study seems to be less efficient than that in BOLERO-2, which can be explained by many reasons. First, this is a real-world observational study. 82.7% patients had visceral metastasis, 60% of them had more than 3 metastatic sites, and 77.3% had received chemotherapy in metastatic settings before everolimus while the corresponding percentage in BOLERO-2 was 58%, 35% and 26%, respectively. The patients’ characteristics in our study are consistent with findings from other real-world studies that oncologists tend to consider the addition of everolimus to endocrine therapy in patients with visceral metastasis, higher tumor burden, and in later lines of treatment [19, 20]. These discrepancies highlight the gap between randomized trials and real-world treatment. The latter indeed reveal more intricate clinical situations in real world, which always trouble clinicians. The results of our study may be a complement especially for those patients with multiple metastases. Second, besides exemestane, everolimus was also combined with anastrozole, letrozole, fulvestrant or tamoxifen in real-world practice. Although lacking robust evidence as BOLERO-2, these regimens have also been proved effective in different settings of clinical trials [21] [22] [14]. There is no direct comparison between these regimens. Exploratory analysis of our study shows that the PFS of everolimus was the longest when combined with exemestane (6.8 months), followed by tamoxifen (5.8months), anatrozole (4.8 months), letrozole (3.2 months) and fulvestrant (3.0 months), although no significant difference was reached. The relative superiority of these combinations still awaits further randomized clinical trials. Third, the median duration of everolimus exposure was only 15.1 weeks in our study, compared with 23.9 weeks in BOLERO-2, which might compromise the efficacy given that the duration of exposure is a surrogate marker for PFS. 14.4% of the discontinuations were up to patients’ choice, mainly because of economic pressure. A cost-effective analysis based on the results of BOLERO-2 shows that the total cost of everolimus/exemestane ($63,584) was 21 times higher than the cost of exemestane alone ($3,010) [23]. Since everolimus hasn't been covered up by Chinese health insurance, it makes sense that people might find it unable to afford.

Exploratory analyses also indicate that patients with symptomatic stomatitis had a significant longer PFS (7.8 vs 4.8months, HR, 0.57, 95%CI 0.34-0.98, *p* = 0.042). Stomatitis is a common adverse event associated with mTOR inhibitors. A meta-analysis of stomatitis incidence of everolimus and its relationship with efficacy included 7 randomized, phase III clinical trials conducted in patients with advanced breast cancer (BOLERO-2 and BOLERO- 3), renal cell carcinoma (RECORD-1), carcinoid tumors (RADIANT-2), and pancreatic neuroendocrine tumors (RADIANT- 3). The occurrence of stomatitis within 8 weeks of everolimus initiation was associated with longer PFS in BOLERO-2 (8.5 vs 6.9 months, HR, 0.78; 95%CI 0.61-1.0), but not in BOLERO-3 studies [24]. The occurrence of specific AEs has been shown to predict better treatment responses both in target and endocrine therapy, such as rash induced by EGFR inhibitors [25, 26], hypertension after sunitinib treatment [27] and arthralgia/myalgia in exemestane treated patients [28]. Mechanism behind these correlations remains unclear. Unpleasant AEs, such as stomatitis or rash, could affect patients’ compliance or lead to discontinuations, which would potentially compromise the efficacy of therapy. The contradictory influence of AE occurrence and dose reduction have on efficacy seem to be paradoxical. Further investigation is required. Howsoever, the potential correlation between symptomatic stomatitis and better efficacy will help improve adherence to everolimus and maximize treatment outcomes [29, 30].

In TAMRAD study, subgroup analysis shows that patients with secondary resistance seem to benefit more from the addition of everolimus than those with primary resistance (CBR in secondary resistance: 74% vs 48%; 46% vs 36% in primary resistance) [14]. In our study, however, everolimus based therapy in patients with primary resistance seemed to be as effective as those with secondary resistance (PFS 5.1 vs 6.1 months, *p* = 0.530).

Despite its striking PFS improvement, everolimus has also aroused many concerns about its toxicities, especially in Asian countries. Because the subgroup analysis of BELORO-2 shows that the incidences of mTOR inhibitor class-effect AEs, such as stomatitis, rash, and noninfectious pneumonitis, were higher among Asian patients [31]. It has become a major concern for oncologists and patients before considering the addition of everolimus in China.

In the present study, with the safety profile consistent with previous studies, the incidences of certain AEs were not as high as expected (Table 5). Stomatitis, the most frequent toxicity experienced with everolimus, is the leading reason for dose interruptions and reductions. In our study, the frequency of stomatitis was 57.1%, similar with 67% in BOLERO-2 [32] and lower than 80% reported in Asian subgroup [31]. One possible reason for the difference was the duration of exposure to everolimus, which was 27.6 weeks in Asian subgroup and 15.1 weeks in our study. Another factor that may attribute to the decrease in stomatitis incidence is the prophylactic measures taken in our study. The prophylactic drugs used in clinical practice include kangfuxin solution and vitamin B group. Kangfuxin solution is a pure Chinese herbal medicine extracted from the American cockroach, and has been proved effective in preventing mucositis induced by chemoradiotherapy in a phase III clinical study of nasopharyngeal carcinoma [33]. Some oncologists also suggest encapsulating everolimus to avoid direct contact. Another study evaluating the effectiveness of these prophylactic measures in preventing the incidence and severity of stomatitis is ongoing in our center. The SWISH trial, recently reported at 2016 ASCO Annual Meeting, proved that the prophylactic use of steroid-based mouthwash markedly decreased the incidence and severity of stomatitis in MBC patients receiving eveolimus/exemesatane, providing another cost-effective way of avoid the dose-limiting toxicity [34].

**Table 5:** Comparison with previous studies

Parameters		FUSCC	BOLERO-2^a^	BOLERO-2
				Asian subgroup^b^
Number of patients	Total	75	485	98
	Safety Population	70	482	98
Age (years)	Median	53	62	59.5
	Range	24-73	34-93	40-79
Exposure duration to everolimus (weeks)	Median	15.1	23.9	27.6
	Range	1-131.6	1-123.3	2-123.3
PFS (months)	Local review	5.9	7.8	8.5
	Central review	NA	11.0	NA
Stomatitis(%)	All grades	57.1	67	80
	≥grade 3	9.3	8	8
Noninfectious pneumonitis (%)	All grades	10	20	13
	≥grade 3	0	4	1
Hyperglycemia(%)	All grades	21.4	16	9
	≥grade 3	1.4	6	4
Hyperlipidemia(%)	All grades	10	14	NA
	≥grade 3	0	1	NA

Abbreviations: PFS, progression-free survival, NA, Not available.

^a^ Survival data from Yardley et al [17]. Safety data from Rugo et al [32].

^b^ Data from Noguchi et al [31].

Noninfectious pneumonitis, a nonmalignant infiltration of the lungs associated with rapamycin derivatives, has a wide spectrum of disease severity varying from subclinical to fulminant, even fatal on rare occasions, therefore aroused many concerns. A recent meta-analysis of five clinical trials (*n* = 2233, 989 had breast cancer) [35] showed that the incidence of all-grade pneumonitis in everolimus 10 mg recipients was 10.4%, similar with 10% reported in our study. Among seven patients with the radiologic evidence of NIP, two patients presented with cough and dyspnea but soon resolved after everolimus discontinuation and short-term steroid therapy. The other five asymptomatic patients continued everolimus treatment. Follow-up CT and careful clinical assessment was performed. None of these patients showed any sign of deterioration.

Hyperglycemia and hyperlipidemia are of special interest because postmenopausal women already are at increased risk for age-related metabolic abnormalities. The incidence for all grade and ≥3 hyperglycemia in our study was 21.4% and 1.4%, compared with 16% and 6% in BOLERO-2 [32]. One limitation of this retrospective study was that not all patients had glucose data before the start of everolimus. Therefore, the incidence was highly likely to be overestimated. The incidence of hyperlipidemia was 10% in the present study, consistent with 14% reported in BOLERO-2 [32].

From the observations from our study, we can see that the AEs of everolimus are manageable, but require physician awareness, patient education and early intervention. Given the time course of everolimus related AEs observed in our study, we recommend a first follow-up visit in 2 weeks after starting everolimus, a laboratory test at least every 2 months, and a thoracic CT scan within 3 months.

This study is of importance for a number of reasons. First, it offers first-hand real-world data of the efficacy and safety of everolimus in combination with endocrine therapy in Chinese patients, which can be informative for clinical oncologists and provide supplementary data for the coming registration clinical trials in China. Second, exploratory analysis provides clues for the selection of patients who are likely to benefit more from the addition of everolimus. Last but not least, safety analysis of this study helps oncologists and patients to gain better knowledge and familiarization with possible side effects and when to expect them. Besides, adverse events of everolimus shown in this study are tolerable and predictable, which gives reassurance to patients who have concerns about toxicities.

This study was a retrospective study, therefore it was subject to limitations including potential missing data, possible recall and information bias and small sample size. Besides, it has been difficult to perform dose-effect analyses due to the complexity of treatment pattern and retrospective nature of this study. Furthermore, quality of life and time to resolution of AEs were not formerly assessed, which could have provided more comprehensive information on everolimus toxicities.

Future efforts to expand the therapeutic benefit of everolimus in patients with HR+ breast cancer include investigating the introduction of everolimus in earlier settings (adjuvant study SWOG: NCT01674140 and UNIRAD: NCT01805271; first line therapy in BOLERO-4), or in different populations (NCT02313051: premenopausal women treated with goserelin after progressed on tamoxifen), or developing new combinations with other inhibition strategies (inhibitors of CDK4-6 (NCT02732119) or PI3K (NCT02077933). In addition, BRAWO, an ongoing large non-interventional study with a planned enrollment of 3000 patients will provide real-world data of everolimus plus exemestane in HR+/HER2- breast cancer. Taken together, we will be able to see the whole picture of the role everolimus plays in the treatment of HR+/ HER2- breast cancer.
